# Risk Factors for Periacetabular Osteolysis and Wear in Asymptomatic Patients with Uncemented Total Hip Arthroplasties

**DOI:** 10.1155/2014/905818

**Published:** 2014-11-16

**Authors:** Buster Sandgren, Joakim Crafoord, Henrik Olivecrona, Göran Garellick, Lars Weidenhielm

**Affiliations:** ^1^Department of Molecular Medicine and Surgery, Karolinska Institutet, 17176 Stockholm, Sweden; ^2^Department of Radiology, Karolinska University Hospital, 17176 Stockholm, Sweden; ^3^Swedish Hip Arthroplasty Register, Registercentrum, 41345 Gothenburg, Sweden

## Abstract

Osteolysis is a silent disease leading to aseptic loosening. This has not been studied in a cohort of asymptomatic patients. The aim of this study was to detect factors that might be associated with the development of periacetabular osteolysis and wear around an uncemented cup. We assessed 206 patients with an uncemented cup, measuring wear and periacetabular osteolysis using computed tomography with a median follow-up of 10 years after surgery (range 7–14 years). EQ5D, pain from the hip, and satisfaction were assessed. The association between periacetabular osteolysis and wear, age, gender, activity, BMI, cup type, cup age, positioning of the cup, and surface coating was investigated with a proportional odds model. Wear and male gender were associated with an increased risk for periacetabular osteolysis. There was no association with periacetabular osteolysis for time from operation, patient age, UCLA Activity Score, liner thickness at time of operation, BMI, cup positioning, and type of implant. A thin liner at time of operation is correlated to increased wear. Linear wear rate was 0.18 mm/year and 46 of 206 patients had large periacetabular osteolysis. Asymptomatic patients with these implants should be followed up on a regular basis with a sensitive method such as CT in order to detect complications early.

## 1. Introduction

Periacetabular osteolysis (PAO) around the artificial hip is a common long-term complication especially in uncemented total hip arthroplasty (THA) with conventional polyethylene liner. Uncemented fixation has often been used in younger patients. Wear products from the polyethylene have been suggested to cause PAO [1–8]. Wear of the polyethylene liner was a common complication in the first generation uncemented cups due to suboptimal sterilization technique, bad locking mechanisms of the liner in the metal shell, and thin polyethylene liners [9, 10]. Patients with these cups have a reported high risk for revision in the Swedish Hip Arthroplasty Register (SHAR). There are usually no clinical symptoms from wear or osteolysis until cup loosening or total liner wear occurs [11]. In a cohort of young asymptomatic patients operated with an implant with a known high revision rate due to wear and osteolysis, severe bone loss can occur before the patents hip symptoms bring him to the doctor. This might result in an extensive revision [12]. If bone loss can be detected early a big reconstruction due to bone loss can be avoided. How shall we follow these patients postoperatively? Can we identify patients at risk for osteolysis and follow them more closely?

Plain radiography is not sufficiently sensitive for detection and description of PAO [13, 14]. CT examination is by far a more accurate method but high radiation levels are a major concern [14]. However the information that can be obtained from CT might outweigh the risk for a higher radiation level compared to conventional radiographic examination.

We have measured wear and PAO in a cohort of asymptomatic patients operated with a total hip arthroplasty with a first generation uncemented cup.

The aim of this study was to analyze risk factors for PAO and wear in this patient cohort.

## 2. Material and Methods

5707 patients younger than 67 years with primary osteoarthrosis operated with an uncemented, metal backed cup with polyethylene liner and metal femoral head between years of 1994 and 2000 were selected from the Swedish Hip Arthroplasty Register (SHAR) (Table 1). Already revised cups were not included in this study.

Eight hundred and twelve of these procedures were performed in Stockholm. They were selected for examination. For consistency reasons we selected those with the five most common cup designs (Table 3), which left us with 395 patients. These patients were contacted by mail and asked if they wanted to participate in the study.

We excluded patients that were under investigation due to suspected cup loosening, inability to understand Swedish, and patients unwilling to participate in the study. This left us with 210 patients. Each patient was offered a clinical examination by one of the authors (B. Sandgren) at a dedicated outpatient appointment where the evaluation forms were filled out. A conventional radiographic examination and a CT scan of their pelvis were done. Four patients did not attend the examinations leaving us with 206 patients (Table 1).

The clinical examination included registration of age, gender, time from implantation to follow-up (cup age), implant type (cup type), EQ-5D, physical activity level assessed with University of California at Los Angeles (UCLA) Activity Score, Body Mass Index (BMI), pain from the investigated hip assessed using the Visual Analog Scale (VAS), and satisfaction with the operated hip assessed with VAS.

Fifty-five out of the 206 cups were bilateral total hip replacements. In cases with bilateral cups, the side with the first operated THR was included and evaluated.

PAO were analysed using inhouse developed dedicated software for orthopaedic analysis that has been presented earlier [15, 16]. PAO was defined as bone loss leading to lack of contact between the pelvic host bone and the metal shell and graded into three levels.

*Grade 1*. The entire cup surface is covered with bone stock. No osteolysis exists.

*Grade 2*. Distance of metal shell with no contact with bone is of less than approximately 10 millimeters (mm). Small osteolysis exists.

*Grade 3*. Distance of metal shell with no contact with bone is of more than approximately 10 mm. Large osteolysis exists.

Linear wear, as opposed to volumetric wear, was measured with a 3D computer program using the outer rim of the head and the inner rim of the cup. The metal head penetration into the liner was measured in millimetres (mm). The distance of linear wear was then divided by the original liner thickness and presented as percentage of linear wear from the original liner thickness. Linear wear was then categorized in to three groups; 1: <29%, 2: 30–49%, and 3: >50%. The method has been tested for validity and repeatability [17] and the error of measurement has been shown to be less than one mm [16].

Cup positioning was calculated from the CT examination follow-up in relation to the plane that includes the superior iliac spines and the pubic tubercles according to McKibbin [18].

In order to investigate the importance of original liner thickness, the original liners were divided into two groups; 1: liner < 6 mm and 2: liner ≥ 6 mm. The two groups were compared for linear wear and PAO.

Original liner thickness was estimated adding head penetration into the liner in mm to liner thickness left. The liners were divided into two groups, 1: liner < 6 mm and 2: liner ≥ 6 mm, and the groups were compared for wear and PAO.

Physical activity level was evaluated using the UCLA Activity Score [19]. The UCLA Activity Score questionnaire was translated to Swedish and back to English and evaluated by an expert group for consistency. 120 patients filled out the UCLA Activity Score questionnaire twice with an interval of four weeks in order to analyze repeatability of the score.

EQ5D was registered on all patients. EQ5D is a standardized, non-disease specific life quality measurement [20].

Present pain on examination from the investigated hip was evaluated using a Visual Analog Scale (VAS) where zero was no pain and ten was unbearable pain.

The level of satisfaction on examination was evaluated using a VAS scale where zero was a totally satisfied patient and ten was a completely unsatisfied patient.

The effect of hydroxy apatite (HA) coating on wear and osteolysis was tested by comparing patients with HA- and non-HA coated Romanus cups (Table 2). The other cup types were of such different designs and were too few to allow for analysis of the effect of HA versus non-HA (Table 3).

It was impossible to determine how each liner was sterilized. We presume that the vast majority of the investigated cups were sterilized with gamma irradiation technique. None of the cup liners had highly crosslinked polyethylene.

Nine variables suspected to correlate to PAO were investigated (Table 4): linear wear (as continuous variable and categorized into 3 groups), cup age (time from implantation to follow-up), patient age at time for operation, gender (two categories), UCLA Activity Score, implant type (three categories: Romanus HA, Romanus non-HA, and others), BMI, estimated liner thickness at time of surgery, and cup position (inclination and anteversion) were investigated.

The study was approved by the ethics committee at the Karolinska Institute (Dnr 2007:1); all of the patients who participated in the study signed an informed consent form.

## 3. Statistics

A proportional odds model was analyzed with PAO as outcome. PAO was categorized in three categories (no osteolysis, small osteolysis, and large osteolysis). A logistic regression with wear as outcome was analyzed. Wear was categorized into two categories (<30% wear and ≥30% wear).

Linearity between the continuous variables and PAO as outcome was investigated.

A proportional odds model with wear as outcome was investigated with the same variables as above both as univariable and as multivariable analysis. Wear was analysed both as a continuous variable and grouped in two categories—more or less than 30% of original liner thickness (Table 5).

Reliability for the UCLA Activity Score was tested with Spearman's correlation and weighted kappa statistics.

The difference in physical activity between overweight patients (BMI ≥ 25) and nonoverweight patients with a BMI less than 25 was tested using Wilcoxon rank sum test.

All the statistical analyses were performed in R v. 2.15.3 (R Foundation for Statistical Computing, Vienna, Austria).

## 4. Results

Our patients were young with a mean age of 53 at operation. The average yearly linear wear was 0.18 mm. Results are presented in Table 2. The median BMI was 27 (range 19–45). There were 68% patients with BMI more than 25, which is defined as overweight [21]. There was no difference in activity level between overweight and nonoverweight patients in this cohort (Wilcoxon's rank sum test, *P* = 0.220) indicating that overweight patients scored their activity at the same level as normal/underweight patients.

Median VAS for pain in the examined hip was 1.2 (range 1–6, only one patient indicated 6) and median VAS for satisfaction had a median of 1.2 (range 1–4) indicating that the patients in this study had little pain and very high satisfaction. However 57 patients had PAO grade 3 and eight patients had more than 50% liner wear; in two of these cases the head had penetrated through the liner into the metal shell.

There was no difference in wear or PAO between the different cups included in the study. Osteolysis and linear wear for each cup type are presented in Table 3.

The univariate analysis showed that wear was the most important risk factor for PAO. Male gender was also identified as a risk factor. Wear was linearly related to PAO but for the other variables there is no association either linear or nonlinear. A multivariate analysis with wear, cup age, patient age at operation, gender, implant type, and original liner thickness did not change the results of the univariate analysis. Age at the time of surgery, cup age, activity level, liner thickness ≤ 6 mm, implant type, and cup position did not show any increased risk for PAO.

The repeatability of the UCLA Activity Score was low with a Spearman correlation coefficient of 0.47 and a weighted kappa coefficient of 0.44 when the patients were reevaluated after four weeks.

The cups were well placed in the acetabulum and only four patients had a cup inclination over 60 degrees. Of these four, the wear was slightly less than average (0.02–0.17 mm/year) and two had no PAO.

We found that wear increased in cups where the original implant liners thickness was less than six mm. The results of factors chosen as possible risk factors for wear are presented in Table 5.

Our main finding in this patient cohort was that wear was the most important factor for increased the risk for osteolysis in asymptomatic patients with first generation uncemented THA. Male gender was also a risk factor for osteolysis but not for liner wear. A thick liner from start decreases the risk for liner wear.

There was no association between UCLA activity score, BMI, Implant type, implant age and patient age and risk of PAO and wear. Recent studies have shown that increased activity can increase the rate of osteolysis [22]. It is surprising that high physical activity was not related to increased wear or PAO in our study. However, this can be explained by our assessment of reliability indicating that the UCLA Activity Score is not a reliable tool to assess physical activity in this patient group, even though it has been frequently used in the literature [23]. One might suspect that a high BMI would increase wear which might lead to increased PAO but we found no association between BMI and wear. We found no evidence in the literature that overweight patients have a higher wear or PAO. One might expect that overweight patients were less physically active leading to less wear and PAO but our data did not indicate an association between low physical activity and high BMI.

The SHAR shows a 10–15% revision rate after 10 years for the five cup types that we selected. The main reason for revision was aseptic loosening due to bone loss. Considering this information, PAO levels were low in our material even though the yearly wear rate was high (0.18 mm) but is within the range of earlier reports [24]. This might be explained by our selection of only asymptomatic patients excluding all the symptomatic and revised cases but it might also be an indication that other factors than wear play a role in developing PAO. However, in light of such a high revision rate and comparably high wear rate, these young patients need to be followed.

The patients in our study did well with very little pain, although 46 patients had a PAO with no contact between the cup and bone of more than approximately 10 mm defined as a large osteolysis in our classification system. Eight patients had a high liner wear but no clinical symptoms. This is in agreement with Lavernias results showing that PAO and wear are an asymptomatic process [11].

A main limitation of this study is that a number of other factors that might influence the development of PAO, such as smoking and the use of corticosteroids, were not assessed.

The selection of cup types is also a limitation in this study. The cups were hemispherical metal shells made of titanium alloy. The coating differed both in design and in content, some with HA coating and some without. The Romanus cup was fixed with a screw technique. It was the most common cup used in Stockholm between 1994 and 2000 and 157 of our 206 investigated cups were of this brand. The Romanus cup has a 15% revision rate after 10 years according to the SHAR, which is comparable to the other cups in this study. However we cannot claim that our results are applicable to all cups from this era.

The patients in this study were operated between 1994 and 2000. We have not been able to get information about the sterilization method for the cup liners, which is a limitation in this study.

Why should we measure distance of lack of contact with the cup instead of volume?

The only reason to measure osteolysis is to estimate the risk of cup loosening. The more the cup is lacking contact with the bone, the greater risk of cup loosening will be.

If you measure the longest distance of osteolysis, you will get a fair estimate of the severity of the osteolytic lesion. The osteolysis is never two-dimensional and a distance multiplied by two will give you a fair estimate of lack of contact between bone and cup.

A pear shaped osteolysis with a large volume but small contact surface with the cup would in our measures be less important than a small volume with a large lack of contact towards the cup surface [25]. You could find an osteolysis with great volume but small contact area to the cup.

The borders of the lesion used when measuring an osteolysis are not well defined, even on high resolution computed tomographies. There is a risk when measuring volume that the measurements are not as accurate as one would hope.

This is to our knowledge the first study assessing PAO in asymptomatic THA patients with uncemented implants. Furthermore, these patients have been examined with CT which is a more sensitive method to detect wear and PAO than conventional radiographic examination [15, 26]. Radiation levels for a single pelvic view are 3.2 mSv which is equivalent to the yearly background radiation levels in Sweden. A conventional radiographic examination including pelvic, frontal hip, and oblique radiographic images is approximately 1.0–1.5 mSv [27]. In order to detect patients with PAO in this patient cohort, regular follow-up examinations are necessary with a sensitive method to assess wear and PAO. We have shown that CT is such a method and is more sensitive than conventional radiographic examination to detect PAO [14]. Other authors have found that early revisions of THR for PAO and wear are beneficial both from the patients' and from the surgeons' stand point and also were cost effective and have suggested that patients should be followed up yearly [11, 12]. We believe that the higher radiation level in the CT examinations is justified by the increased ability to detect PAO.

In conclusion, we found that wear was associated with PAO. Since this is an asymptomatic process that may lead to aseptic loosening, we recommend that patients with these implants should be followed up on a regular basis with a sensitive method such as CT in order to detect complications early.

## Figures and Tables

**Table 1:** Patient selection.

Patients with uncemented cups in Sweden operated between 1994 and 2000	5707
Patients were operated in the region of Stockholm	812
Patients excluded according to the exclusion criteria: posttraumatic arthrosis, deceased, waiting for, or under investigation for, revision surgery.	
The five most common cups were selected	395
Patients chose not to participate	185
Patients that failed to attend	3
263 cups in 206 patients. Only one hip/patient was evaluated	
206 patients were included in this study	

**Table 2:** Results.

Male/female	93/113
Years at operation median (range)	53 (19–67)
Side right/left	107/99
Years from operation, median (range)	10 (7–14) SD 2.12
Uni/bilateral	151/55 = 206 investigated cups
Wear at median 10 years	
Median (range)	1.8 (0.3–9.0) SD 0.4
Wear/year, median (range)	0.18 mm (0.02–1.26) SD 0.16
EQ5D, median (range)	1 (0.6–1.0)
VAS pain, median (range)	1 (1–4)
VAS satisfaction, median (range)	1 (1–3)
UCLA, median (range)	6 (2–10)
Anteversion degrees, median (range)	19 (−30–58)
Inclination degrees, median (range)	42 (2–70)
Cups with >60-degree inclination	4

**Table 3:** Osteolysis and wear for each cup type. Osteolysis classified according to the system presented in the text. Wear presented as categorical data: (1) 0–29%, (2) 30–49%, and (3) >59.

Cup	Number	Osteolysis			Wear		
		1	2	3	1	2	3
Romanus HA^*^	82	14	51	17	57	19	5
Romanus non HA^*^	75	12	42	21	54	22	1
ABG HA^**^	27	1	22	4	22	5	0
Omnifit^**^	16	2	11	3	10	2	2
Trilogy^***^	6	0	5	1	5	1	0

^*^Biomet Orthopaedics, Warsaw, Indiana, USA.

^**^Stryker, Hopkinton, Massachusetts, USA.

^***^Zimmer, Warsaw, Indiana, USA.

**Table 4:** Periacetabular osteolysis as outcome-proportional odds model.

Variables	Univariable			Multivariable with wear as categ. data <30% and ≥30%		
	Odds ratio	95% confidence interval	*P* value	Odds ratio	95% confidence interval	*P* value
Wear (head penetration into the liner in mm)^*^	1.4	1.1–1.8	0.002	2.1	1.1–3.9	0.028
Cup age	1.1	0.9–1.2	0.452	1.0	0.9–1.2	0.623
Patient age at time of surgery^*^	0.9	0.6–1.4	0.770	1.0	0.7–1.5	0.996
Male versus Female	1.8	1.0–3.1	0.042	1.8	1.0–3.2	0.045
UCLA Activity Score	1.0	0.9–1.2	0.827			
Cup type categ.: Romanus HA/Romanus non-HA/other cups	1.1	0.6–2.1	0.649	1.1	0.6–2.1	0.714
Liner ≥ 6 mm versus <6 mm	0.9	0.5–1.7	0.724	1.0	0.5–2.0	0.979
BMI^*^	1.0	0.8–1.6	0.931			
Anteversion^*^	1.3	0.8–2.1	0.383			
Inclination^*^	1.2	0.9–1.6	0.309			

^*^Modelled as continuous variables but presented in units of ten.

**Table 5:** Logistic regression model with wear as outcome.

Variable	Univariable			Multivariable		
	Odds ratio	Confidence interval	*P* value	Odds ratio	Confidence interval	*P* value
Cup age at time for investigation	1.2	1.0–1.4	0.062	1.3	1.0–1.5	0.016
Patient age at operation	0.9	0.6–1.4	0.690	0.9	0.8–3.0	0.753
Male versus Female	1.3	0.7–2.4	0.407	1.6	0.8–3.0	0.190
UCLA	1.0	0.9–1.3	0.742			
Original liner^*^ contin. data	0.1	0.0–0.4	0.001			
Original liner categ. < 6 mm	0.3	0.2–0.7	0.003	0.3	0.1–0.6	0.001
BMI^*^	1.1	0.6–2.1	0.650			
Anteversion^*^	1.0	0.8–1.3	0.686			
Inclination^*^	1.3	0.9–1.8	0.156			

^*^Modelled as continuous variables but presented in units of ten.
